# Exchange Bias Effect of Ni@(NiO,Ni(OH)_2_) Core/Shell Nanowires Synthesized by Electrochemical Deposition in Nanoporous Alumina Membranes

**DOI:** 10.3390/ijms24087036

**Published:** 2023-04-11

**Authors:** Javier García, Ruth Gutiérrez, Ana S. González, Ana I. Jiménez-Ramirez, Yolanda Álvarez, Víctor Vega, Heiko Reith, Karin Leistner, Carlos Luna, Kornelius Nielsch, Víctor M. Prida

**Affiliations:** 1 Departamento de Física, Facultad de Ciencias, Universidad de Oviedo, C/Federico García Lorca 18, 33007 Oviedo, Spain; garciafjavier@uniovi.es (J.G.); gonzalezgana@uniovi.es (A.S.G.);; 2 Laboratorio de Membranas Nanoporosas, Edificio de Servicios Científico Técnicos “Severo Ochoa”, Universidad de Oviedo, C/Fernando Bonguera s/n, 33006 Oviedo, Spain; vegavictor@uniovi.es; 3 Leibniz Institute for Solid State and Materials Research (IFW) Dresden, Helmholtzstraße 20, 01069 Dresden, Germany; h.reith@ifw-dresden.de (H.R.); karin.leistner@chemie.tu-chemnitz.de (K.L.); k.nielsch@ifw-dresden.de (K.N.); 4 Electrochemical Sensors and Energy Storage, Faculty of Natural Sciences, Institute of Chemistry, TU Chemnitz, Strasse der Nationen 62, 09111 Chemnitz, Germany; 5 Facultad de Ciencias Físico Matemáticas (FCFM), Universidad Autónoma de Nuevo León (UANL), Av. Universidad S/N, San Nicolás de los Garza 66455, Nuevo León, Mexico; carlos.lunacd@uanl.edu.mx

**Keywords:** nanoporous alumina membranes, nanowires, core/shell, antiferromagnetism, exchange bias, FORC

## Abstract

Tuning and controlling the magnetic properties of nanomaterials is crucial to implement new and reliable technologies based on magnetic hyperthermia, spintronics, or sensors, among others. Despite variations in the alloy composition as well as the realization of several post material fabrication treatments, magnetic heterostructures as ferromagnetic/antiferromagnetic coupled layers have been widely used to modify or generate unidirectional magnetic anisotropies. In this work, a pure electrochemical approach has been used to fabricate core (FM)/shell (AFM) Ni@(NiO,Ni(OH)_2_) nanowire arrays, avoiding thermal oxidation procedures incompatible with integrative semiconductor technologies. Besides the morphology and compositional characterization of these core/shell nanowires, their peculiar magnetic properties have been studied by temperature dependent (isothermal) hysteresis loops, thermomagnetic curves and FORC analysis, revealing the existence of two different effects derived from Ni nanowires’ surface oxidation over the magnetic performance of the array. First of all, a magnetic hardening of the nanowires along the parallel direction of the applied magnetic field with respect their long axis (easy magnetization axis) has been found. The increase in coercivity, as an effect of surface oxidation, has been observed to be around 17% (43%) at 300 K (50 K). On the other hand, an increasing exchange bias effect on decreasing temperature has been encountered when field cooling (3T) the oxidized Ni@(NiO,Ni(OH)_2_) nanowires below 100 K along their parallel lengths.

## 1. Introduction

Nanostructured materials have been widely investigated due to the eagerness of finding novel phenomena that can potentially be implemented in the development of new technological devices as well as in the improvement of existing ones [1,2,3]. When decreasing the size of a material down to the nanometre scale, both quantum and surface effects might become dominant, leading to new properties or transport phenomena. In the case of magnetic nanomaterials, size reduction is also accompanied by stronger and tuneable magnetic shape anisotropy, which opens up a wide range of opportunities to control magnetization reversal processes. In applications nowadays, magnetic materials are widely used in data storage, energy conversion or spintronics, among others [4,5]. In some cases, very stable magnets are required to avoid information losses, which require the highest possible coercivity and remanence values. However, if information has to be written, the coercive field must not exceed the maximum field value that can be applied by the writing head. This fact reveals the need for tuneability of the material magnetic properties in a fine and controlled manner. In nanomaterials, this has been achieved by proper selection of the material alloy composition and fabrication methods that lead to an adjustment of magnetic properties through a suitable combination of shape and magnetocrystalline anisotropies [1,6,7,8,9]. On the other hand, spintronics commonly requires several magnetic layers to achieve different parallel/antiparallel configurations of spins between separate layers [10,11,12]. In this case, not only a defined magnetization reversal in terms of coercive field and remanence (uniaxial magnetic anisotropy) is needed. In fact, considering that magnetic layers are usually submitted to the same applied magnetic field, to achieve different magnetization configurations, they must be magnetically decoupled by introducing a unidirectional anisotropy. Usually, this can be accomplished by a proper engineering of the exchange coupling of spins at a ferromagnetic (FM)/antiferromagnetic (AFM) interface [12,13,14,15]. Depending on the magnetic anisotropy of the AFM layer, two main phenomena can occur if one considers a perfect interface. First of all, if the anisotropy of the AFM layer is low, its magnetization can be reoriented to align the spins at the interface with the magnetization reversal of the FM layer, yielding to an increase in the coercive field of the whole system. On the other hand, if the anisotropy of the AFM layer is high enough, it is not altered by an external magnetic field or a magnetization reversal of the FM layer, giving as a result a shift of the FM hysteresis loop along the field axis, which is commonly denoted as the Exchange Bias (EB) effect [12,13]. Whether one effect or another takes place is directly related to the quality of the AFM layer as well as the interface. In fact, if the AFM layer presents granular (structural, morphological or magnetic) behaviour, both effects could be observed with the same sample. Such microscopic magnetic properties of multiferroic materials and their functional applications are strongly dependent on the size, spatial arrangement, and morphology of the magnetic domain configuration [16]. The magnetic domains structure of Ferromagnetic (FM) materials is based on minimization of magnetostatic interactions, while in antiferromagnets, or similar magnetic materials exhibiting a compensated magnetic ordering due to their zero net magnetization, the magnetic domain formation cannot be strictly attributed to the effect of demagnetizing field [17]. While extrinsic phenomena such as magnetoelastic interactions derived from tensile-strain profiles, grain size distribution or thermal annealing procedures can control the configuration of peculiar magnetic domains, other intrinsic, and not fully understood, features are also competing to determine the size and shape of domain distribution [18]. Thus, a clear identification and control of the intrinsic features that govern the magnetic domain configuration of an antiferromagnet is therefore required.

The EB effect is the best indicator for the existence of a unidirectional magnetic anisotropy, which can be achieved by a high orientation of the AFM layer. To achieve this, the sample must be field cooled below the Neel’s temperature (T_N_) of the antiferromagnet. In order to use this property of AFM, a T_N_ well above room temperature (RT) is of great interest to develop systems operating at ambient conditions. This is one of the main reasons why NiO is such an interesting AFM material, since its bulk T_N_ is reached at around 525 K, in addition to its undeniable industrial potential due to its abundance, chemical stability and, in most cases, cost-effective fabrication [19,20]. On the downside, as a result of the size reduction of the AFM nanomaterials, finite size effects have been observed in AFM nanoparticles, yielding to the appearance of a blocking temperature, T_B_, that suppresses the EB effect well below T_N_ [20,21,22,23,24,25,26,27]. Furthermore, the production and study of well-defined and homogeneous core (FM)/shell (AFM) nanowires is still demanded [13,28,29,30,31]. It should be noted that other types of materials where Ni is also in the Ni^2+^ oxidation state can show interesting magnetic phenomena at the nanoscale. This is the case with Ni(OH)_2_, which can crystalize into two different pseudopolymorphic states denoted as α- and β-Ni(OH)_2_, respectively. While both phases crystallize into a hexagonal structure, the main difference is that the α-Ni(OH)_2_ includes intercalated anions and water molecules [32,33]. Although both phases are formed by stacked layers along the c-crystallographic axis, the α-Ni(OH)_2_ shows typically larger c-axis size depending on the hydration level. The magnetic properties and the type of magnetic ordering exhibited by these two phases is still a subject of controversy and the available literature shows discordant results [32,33,34,35,36]. It has been proposed that, for short c-parameters (β-Ni(OH)_2_) the exchange interaction among Ni atoms of the same a-b plane layer is ferromagnetic, while an antiferromagnetic coupling is expected between layers stacked along the c-axis direction. As exchange interaction is more dependent on interatomic distances, the hydration level that changes the c-parameter in α-Ni(OH)_2_ is expected to have a strong impact on the magnetic ordering of Ni(OH)_2_ materials [32].

In this work, the magnetic properties of arrays of core (FM)/shell (AFM) Ni@(NiO,Ni(OH)_2_) nanowire arrays are studied. These nanowires (NWs) have been fabricated by means of purely electrochemical deposition methods compatible with standard microelectronics technology, where nanoporous alumina membranes (NAMs) have been used as patterned templates. An electrochemical process has also been used to oxidize the Ni nanowires surface in a controlled and reversible manner. In order to investigate the impact of the fabrication process as well as the cylindrical nature of the nanowires over the magnetic properties of the FM/AFM interface, temperature-dependent hysteresis loops (HL) and thermomagnetic ZFC-FC-FH curves have been measured on samples at different stages of the fabrication process. Finally, the first-order reversal curve (FORC) method is employed with the aim of finding the different contributions and magnetic interactions of FM and AFM layers of the NWs in the array on their overall magnetic behaviour. Besides the interesting magnetic properties exhibited by these core/shell nanowires formed by a FM Ni core, covered with the AFM shell of Ni oxides and hydroxides, these nanostructured materials have also recently demonstrated exciting energy storage abilities as novel supercapacitors, which reveal their multifunctional properties for many technological applications.

## 2. Results

### 2.1. Fabrication and Characterization of Ni@(NiO,Ni(OH)_2_) Core/Shell Nanowires

**Figure 1:**
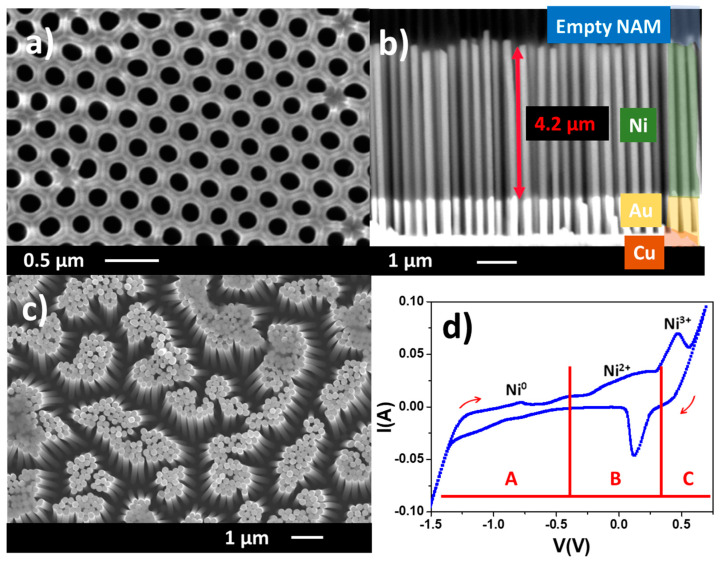
(**a**) Top-view SEM image of the NAM template and (**b**) cross-section view of the Ni nanowires array. (**c**) Top view of crowd Ni nanowires array after NAM removal. (**d**) Cyclic voltammetry curves of the Ni nanowires array sample; A, B and C regions mark off the different oxidation states. Red arrows highlight the respective ascendant and descendant branches of the cyclic voltammetry.

The specific fabrication procedure of the core/shell Ni-based NWs is detailed in Section 4. In short, after the template-assisted electrodeposition of Ni nanowires, the NAM used as a template in the process is selectively etched to expose the Ni nanowire’s surface for electro-oxidization in 1M KOH. From SEM images shown in Figure 1a,b, the fabricated Ni nanowires display a mean diameter of around 170 nm and 4.2 µm in length. One important fact of this study involves the removal of the NAM template to expose the Ni nanowires surface to the oxidating electrolyte. In Figure 1c, the top-view SEM image of the resulting sample (resembling a “nanowires forest”), demonstrates that, as the nanowires do not have the supporting NAM, they tend to crowd together, which must be considered also in the interpretation of the magnetic measurements. Nevertheless, these samples were studied electrochemically to oxidize in a controlled manner the surface of the nanowires and therefore create the core/shell nanostructures. 

The cyclic voltammetry (CV) study carried out on a nickel nanowires sample can be divided into three regions [37], as observed in Figure 1d. In the transition from region A to B (following red arrows), an anodic curve (positive current) is observed, which can be ascribed to the oxidation of metallic nickel. This transition from oxidation states Ni^0^ to Ni^2+^, constitutes the region of interest for this work as the surface of Ni turns into α-Ni(OH)_2_. Further increase in the applied voltage, transiting from region B to C, causes the oxidation of Ni^2+^ to Ni^3+^ to form the NiOOH phase. When the voltage is decreased back to negative values, the reduction reactions occur and the Ni surface turns back to Ni(OH)_2_, but in the β-Ni(OH)_2_ phase. From the CV mentioned above, to oxidize the Ni nanowires surface in a smooth and controlled way into Ni(OH)_2_, it is necessary to apply a voltage to the electrochemical cell whose value falls in the working window region B. In order to slow down the reaction, avoiding potential damage or mechanical stresses to the sample, voltage ramp was applied starting at an initial potential of −0.9 V (Ni) followed by 30 mV/s voltage ramp to reach −0.25 V. This potential was kept constant for 20 min, ensuring the oxidation reaction of the entire nanowire surface. 

**Figure 2:**
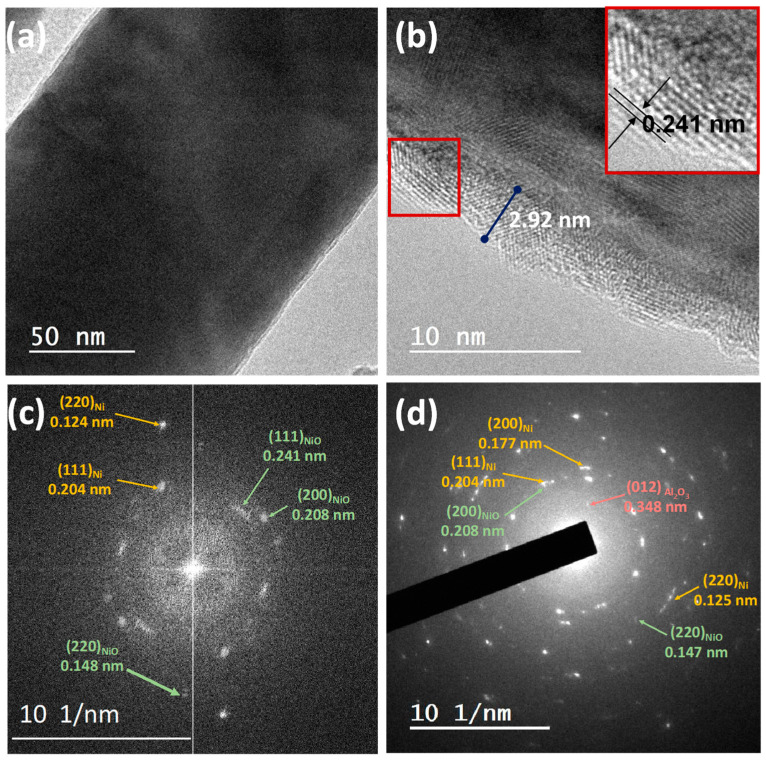
TEM and SAED studies of untreated Ni nanowires: (**a**) High-Magnification TEM image. (**b**) High-Resolution TEM image. The inset image is a magnification of the most external part of the nanowire where lattice fringes associated with the {111} planes of NiO are observed. (**c**) FFT of the micrograph of panel (**b**). (**d**) SAED pattern of the same nanowire.

Figure 2a shows a High-Magnification TEM image of a section of an untreated Ni nanowire. A very thin coating layer with less contrast than the Ni nanowire core can be observed. This lighter outer layer could be associated to the presence of an oxide shell with lower density than the metallic core of the Ni nanowire, and therefore exhibiting lower electron absorption, whose average thickness value is approximately 3 nm (Figure 2b). In agreement with the presence of those two phases, lattice fringes corresponding to both crystalline phases were observed in the High-Resolution TEM (HR-TEM) images (Figure 2b). The measurements of interplanar spacings in their associated Fast Fourier Transforms (FFT), shown in the Figure 2c, provide values of 0.204 nm, 0.177 nm, and 0.125 nm associated with the metallic nickel face-centred cubic phase (JCPDS Card No. 04-0850), and 0.241 nm, 0.208 nm, together with the interplanar distance of 0.148 nm, associated with the face-centred cubic phase of nickel oxide (JCPDS Card No. 04-0835). These same values were measured in the SAED patterns (Figure 2d). Moreover, some additional reflections were also detected and associated with the presence of residues of the reaction medium and alumina. As an illustrative example, a diffraction spot with an associated d-spacing of 0.348 nm is observed, which can be ascribed to the {012} planes of Al_2_O_3_ (JCPDS Card No 46-1212) (Figure 2d).

**Figure 3:**
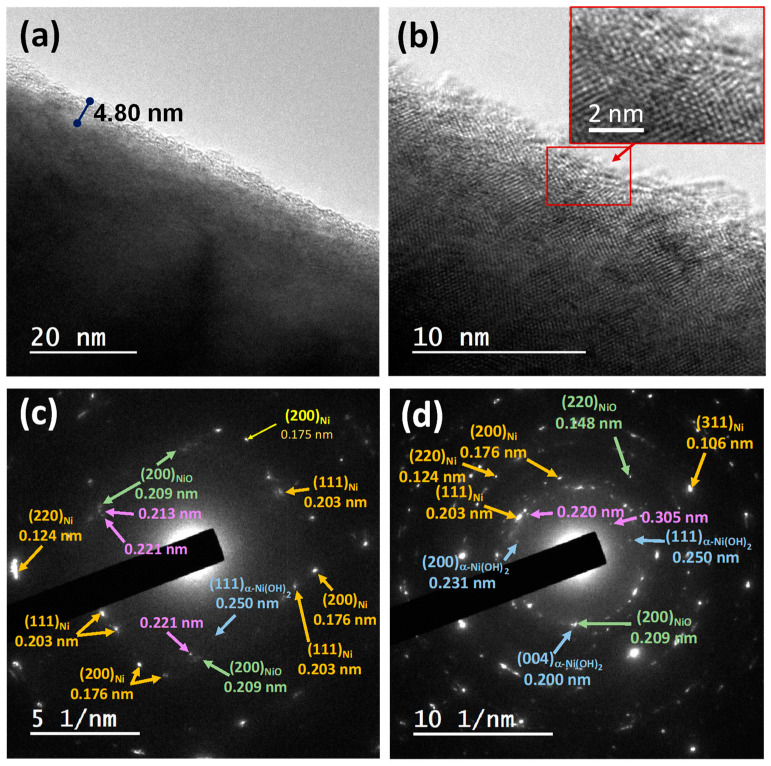
TEM and SAED studies of surface-treated Ni nanowires: (**a**) High-Magnification TEM image. (**b**) High-Resolution TEM image. (**c**,**d**) SAED patterns of different regions of the same nanowire.

The High-Magnification TEM images also revealed a core/shell structure for the treated Ni nanowires, observing a thin surface layer of around 4–5 nm in thickness (Figure 3a). The HRTEM images obtained at the border of the nanowires (Figure 3b) depict a surface with high structural irregularity that exhibits a high density of crystal defects and small crystalline domains of few unitary cells. The SAED patterns of these nanowires display diffraction spots that can be ascribed to the cubic phases of metallic Ni and NiO (Figure 3c,d). Some additional spots appear with associated d-spacings around 0.200 nm, 0.231 nm and 0.250 nm. These spots are in good agreement with the formation of the α-Ni(OH)_2_ phase [32]. Moreover, other spots with undetermined d-spacings of 0.213 nm, 0.220 nm and 0.305 nm are also detected and could be due to residues of the reaction medium and superlattices due to stacking faults [38,39]. 

**Figure 4:**
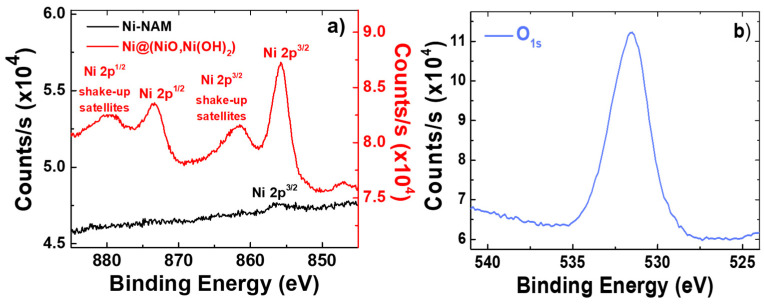
(**a**) XPS analysis of both samples, the former Ni-NAM nanowires (black), and electrochemical treated Ni@(NiO,Ni(OH)_2_) nanowires (red), to the Ni2p core level spectra; (**b**) XPS analysis of the Ni@(NiO,Ni(OH)_2_) nanowires sample to the O1s core level spectra (blue).

In order to obtain detailed information regarding the surface chemistry of the nanowire samples before and after surface electrochemical treatment, an XPS analysis was performed. Figure 4a) shows the Ni^2p^ core level spectra for the two different Ni NW samples. The obtained Ni2p spectrum for the as-fabricated sample (Figure 4a, in black) exhibits only a single weak peak at the energy binding of 856.1 eV, which is in good agreement with previously published XPS data for Ni [40,41]. However, the sample whose surfaces have been electrochemically treated presents two intensive peaks (Figure 4a, in red), which are ascribed to the values of Ni2p_3/2_ (855.6 eV) and Ni2p_1/2_ (873.1 eV) spectra, and two other additional peaks that correspond to their satellites appearing at the binding energies of 861.7 and 880.1 eV. All these values are in good agreement with previously published XPS data for Ni(OH)_2_. [40,41]. The Ni–OH bond is also detected at O1s (peak observed at 531.4 eV, in blue), as shown in Figure 4b) [40,41,42,43]. Therefore, it is clear that after the specific electrochemical process to which the former Ni NWs were submitted, a hydroxide surface layer consisting of Ni(OH)_2_ has been developed covering the external shell of the Ni nanowires. In addition, from HR-TEM characterization, a NiO layer is still detected, which is in good agreement with the formation of a NiO/Ni(OH)_2_ bilayer after the electrochemical oxidation process [32]. 

### 2.2. Magnetic Characterization of Ni@(NiO,Ni(OH)_2_) Nanowires

**Figure 5:**
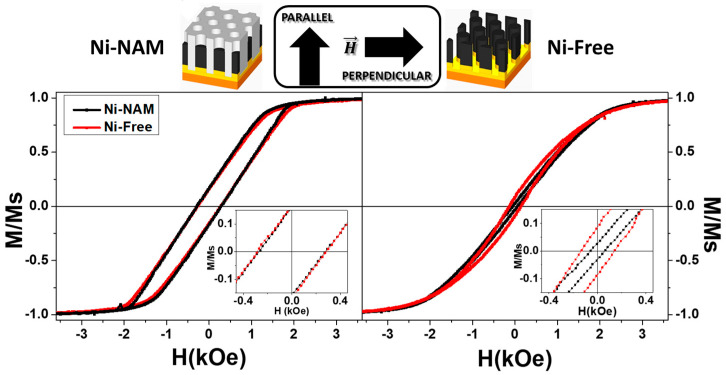
Magnetic hysteresis loops along the parallel (**left**) and perpendicular (**right**) direction of the applied magnetic field respect to NWs axis for Ni-NAM (black) and Ni-Free (red) samples. For reasons of clarity, drawings indicating sample type and direction of the applied magnetic field are also included.

Taking into account that the main characterization method of magnetic properties involves the measurement of a macroscopic piece of the nanowire array, it becomes necessary to study the impact of removing the alumina template from the sample. In order to rule out any change produced by this process and not by the electrochemical treatment itself, room-temperature hysteresis loops (HL) of two different samples with (Ni-NAM) and without (Ni-Free) NAM are compared in Figure 5 under two different orientations of the applied magnetic field, parallel or perpendicularly applied to the nanowires’ longitudinal axis. As a reference, the sample (Ni-NAM) with Ni nanowires still confined into the pores of the alumina template corresponds to the case in which the nanowires are not oxidized (only the FM behaviour is present), and their axial orientation is well defined. On the other hand, the Ni-Free sample corresponds to the nanowire array after the NAM has been removed, but nanowires are not yet electrochemically treated. As can be seen in Figure 5, the Ni-NAM sample shows a clear uniaxial anisotropy coming mainly from the magnetic shape anisotropy with its easy magnetization axis pointing along the parallel direction with respect to the nanowires’ long axis. In fact, along this direction, the removal of the NAM does not have a significant impact on the magnetic behaviour of the Ni-Free sample. 

Only the perpendicular HL of Ni-free samples shows a broadening around the coercive field, which can be produced due to some orientation of the nanowires (and thus an orientation of their easy magnetization axis), along the perpendicular direction as they are free at one end. What it is clear from these results is that all samples can be studied and compared along the parallel direction since the axial orientation of the nanowires is maintained and the exhibited magnetic behaviour does not depend on the NAM removal.

**Figure 6:**
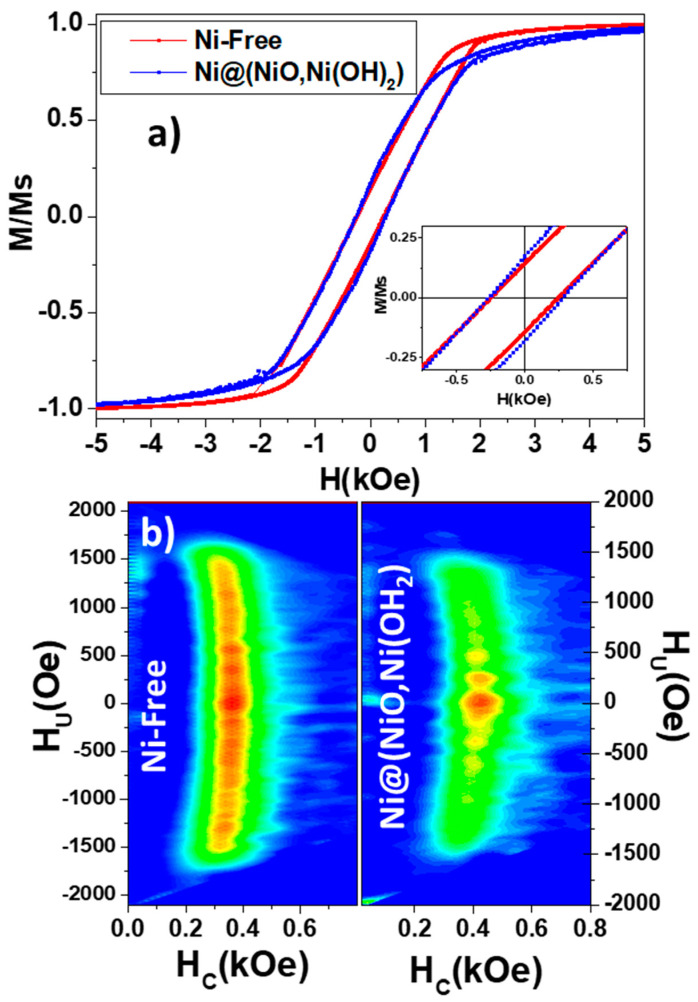
(**a**) Room-temperature magnetic hysteresis loops of Ni-Free (red) and Ni@(NiO,Ni(OH)_2_) (blue) samples with the magnetic field applied along the nanowire’s long axis. (**b**) FORC diagrams of Ni-Free (**left**) and Ni@(NiO,Ni(OH)_2_) (**right**) samples.

Although it has been stated before that samples show a clearly well-defined uniaxial magnetic anisotropy, the shape of parallel HLs needs further discussion and interpretation. These HLs show a tilting along the magnetic field axis by increasing the field at which the magnetization saturates while keeping their width constant. This effect is typical from a system submitted to magnetostatic interactions among particles. In order to extract the maximum information from these measurements, it is necessary to decouple the contribution from the individual nanowires and the magnetostatic interaction. It is in this aspect where FORC distributions analysis plays an important role in the current magnetic characterization of nanomaterials. When comparing the parallel HLs displayed in Figure 6a for both the Ni-Free NWs and the NWs sample that has been electrochemically oxidized (Ni@(NiO,Ni(OH)_2_)), it is possible to observe some differences, although very subtle ones. First, around saturation magnetization, the HL of Ni@(NiO,Ni(OH)_2_) sample shows more curvature closing up its branches, indicating greater contribution of reversible processes. In fact, when comparing the FORC diagrams shown in Figure 6b, the distribution along the Hu axis is diffused for the Ni@(NiO,Ni(OH)_2_) sample showing a more prominent distribution of non-interacting particles. This effect can also be observed in the HLs since the treated sample shows a reduction in tilting at field values near coercivity. This trend could be ascribed to the reduction in the saturation magnetization or some shielding of the magnetic straight lines due to the antiferromagnetic NiO/Ni(OH)_2_ shell. Paying attention at the position of the FORC distribution along the Hc axis, the mean coercive field of Ni@(NiO,Ni(OH)_2_) sample has increased around 17% with respect to the mean coercive field shown by Ni-Free FORC distribution. Taking these facts into account, it seems that at room temperature, the oxidation of the Ni nanowires’ surface provokes a magnetic hardening of the nanowires along the parallel direction, which could be compatible with a low anisotropic AFM layer. It is also worth mentioning the differences between the coercive field of the hysteresis loop and the one obtained with the FORC method. Nevertheless, it can be well understood as the coercivity of the HL only expresses the magnetic field value at which the net magnetization of the whole magnetic system becomes zero [44]. On the contrary, the FORC method allows extraction of a value closer to the switching field of every magnetic element (nanowire) in the system. 

**Figure 7:**
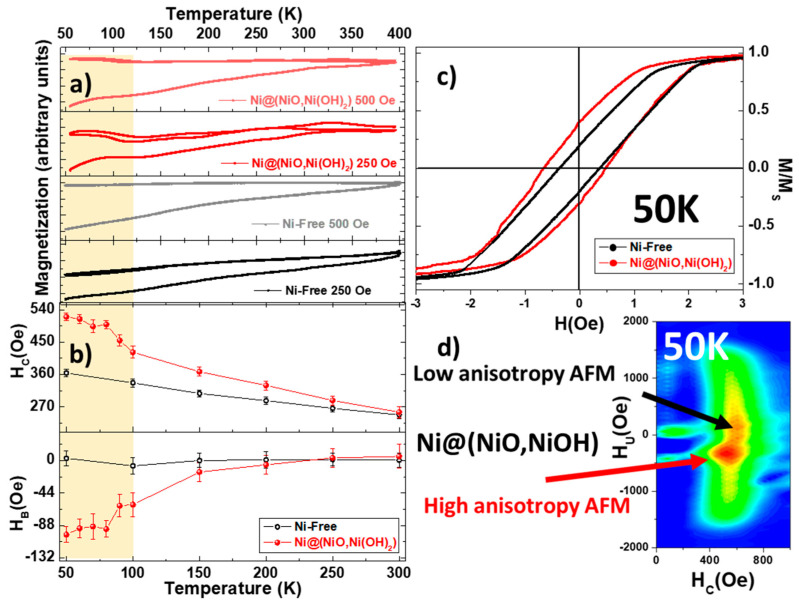
(**a**) Thermomagnetic ZFC-FC-FH curves of Ni-Free (black) and Ni@(NiO,Ni(OH)_2_) (red) samples, measured at the two magnetic field values of 250 Oe and 500 Oe parallel applied to NWs longitudinal axis. (**b**) Exchange Bias field (H_B_) and coercive field (H_C_) obtained from parallel HLs measured along the NWs axis as a function of temperature after field cooling protocol. (**c**) Comparison of the parallel HLs measured for both samples at 50 K and (**d**) FORC diagram of Ni@(NiO,Ni(OH)_2_) sample measured at 50 K.

However, the best fingerprint of an AFM material considering an FM/AFM coupled system is the presence of the Exchange Bias, EB, effect. As is well known, for the E_B_ effect to appear, it becomes necessary to orient the magnetization of both FM/AFM layers at the interface by cooling the sample below the Neel’s temperature, T_N_, of the AFM layer under an external applied magnetic field [12,45]. In the Figure 7a, ZFC-FC-FH curves are plotted for different values of the magnetic field applied along the parallel direction to the NWs length axis. For the Ni-Free sample, a typical magnetic behaviour corresponding to magnetic monodomain nanoparticles can be observed, with blocking temperature, T_B_, above room temperature [46,47]. However, once the samples are electrochemically oxidized, a new feature can be observed below 100 K. Besides the contribution of the pure Ni core, a blocking temperature coming from the AFM layer (only observed in Ni@(NiO,Ni(OH)_2_)-treated samples) can be seen. Although, as has already been mentioned, thr T_N_ of bulk AFM NiO is slightly above 500 K, a reduction in this value below 100 K is commonly found in the literature with a reduction in size of the nanoparticles [20,21,22,23,24,25,26,27]. On the contrary, the T_N_ of Ni(OH)_2_ is typically in the range of tenths of Kelvin [32]. Measuring the parallel HL after a cooling down with an applied magnetic field of 3T along the same direction, a shift as well as a broadening of the loops are encountered, as can be perceived in Figure 7b,c. In fact, both the shift and the coercive field increasing effects are only present at temperatures below 100 K on the Ni@(NiO,Ni(OH)_2_) thermomagnetic curve, bearing out the presence of an AFM shell with a blocking temperature of around 100 K.

Finally, paying particular attention to the FORC diagram displayed in Figure 7d, the convolution of two different FORC distributions can be clearly distinguished. The first question that arises concerns the origin of these two distinct behaviours. Although there is not an easy answer, it is reasonable to think that the statistical dispersion of nanowires’ lengths and diameters may be correlated. Furthermore, the electrochemical oxidation may produce different NiO/Ni(OH)_2_ shell thicknesses and/or morphology in different nanowires. Thus, these different behaviours could be ascribed to two different families of nanowires that behave magnetically differently. One of the FORC distributions presents a rounded shape shifted to negative values of the interaction field H_U_ axis (see red arrow in Figure 7d. This might correspond, as it presents a strong EB effect, to Ni@(NiO,Ni(OH)_2_) core/shell nanowires with a highly anisotropic AFM layer. The other contribution to the overall FORC diagram, highlighted with a black arrow, corresponds to a distribution elongated along the H_U_ axis centred at H_U_ = 0 Oe. This type of distribution corresponds to a homogeneous magnetization reversal mechanism submitted to magnetostatic interaction among particles and not a net shift in the magnetic field axis due to any EB effect [48,49]. However, this distribution is centred at higher values of the coercive field, which can be correlated with the case of a low anisotropic AFM layer producing a magnetic hardening of the FM/AFM coupling [13].

## 3. Discussion

As a result of the study presented above, after the electrochemical oxidation of electrodeposited Ni nanowire surfaces, two different effects have been observed in the core/shell Ni@(NiO,Ni(OH)_2_) nanowires. First, from FORC analysis, a magnetic hardening of the core/shell nanowires has been observed with an increase of around 17% in coercivity at room temperature with respect to the non-treated sample. As an alternative to overcome the lack of shape anisotropy, this effect has also been pursued in FM/AFM nanoparticles to increase their energy production. This magnitude is frequently used to characterize the magnetic energy stored in a magnetic material, and it is calculated as the maximum value of the $\left|B\cdot H\right|$ product in the demagnetizing region of a B−H curve. Furthermore, an EB effect is observed within an applied magnetic field along the parallel direction with respect to the nanowires’ long axis. In fact, excluding the thin film technology and comparing with state of the art of FM/AFM nanoparticles, competing values of EB have been achieved for temperatures as high as 100 K. However, future research must be focused on the study of this effect in single nonentities in order to obtain the precise information that opens the door to optimize the FM/AFM exchange coupling in these cylindrical systems. In the particular case of these core/shell nanowires, it is expected that the magnetic frustration of the antiferromagnetic (AFM) coupling occurs in the NiO and nickel hydroxide (Ni(OH)_2_) phases that appear in the nanometric oxidized outer-shell layer with the crystalline domains of few unitary cells due to their low dimensionality [50]. At the same time, the small mismatch between the lattice parameters of both coexisting crystal phases appeared in the outer shell could lead to a large amount of uncompensated magnetic moments, resulting in an enhancement of the exchange bias effect [51]. Furthermore, similar core/shell nanostructures have recently revealed strong anisotropic magnetic behaviour that is strongly dependent on the specific thermomagnetic conditions of the field-cooling protocol, giving rise to well-defined magnetization states of the AFM outer shell, displaying high-field magnetic irreversibility during the magnetization reversal process, which can be attributed to an AFM easy magnetization axis reorientation, allowing for tuned high-field reversal processes in exchange-biased core/shell nanomaterials [52]. On the other hand, these AFM phases appeared at the outer shell layer of the core/shell Ni@(NiO,Ni(OH)_2_) nanowires, presenting a high density of crystalline defects (dislocations, vacancies and stacking faults) and probably deviations from their stoichiometry. It is well known that both factors, the frustration of magnetic interactions and structural disorder at nanoscale, can produce spin-glass like magnetic behaviours in the AFM surfaces [53,54]. The possibility of simultaneous occurrence for all these novel magnetic phenomena in nanoscale materials still remains as an open question to be investigated.

## 4. Materials and Methods

### 4.1. Fabrication of Ni Nanowires 

Ni nanowires were synthesized by employing prepatterned nanoporous alumina membranes, NAMs, as templates through well-known electrochemical deposition processes [55]. In this regard, high-purity Al foils (99.999%, SMP, Barcelona, Spain) were employed as starting substrates for the synthesis of such NAMs [56,57]. The substrates were cleaned in ethanol and isopropanol, and electropolished in a mixture of perchloric acid and ethanol (1:3 vol.%) under 20 V and vigorous stirring for 9 min. Afterwards, the Al substrates were submitted to a hard anodization (HA) process carried out in 0.3 M oxalic acid electrolyte containing 5 vol.% of ethanol as antifreeze agent, at a temperature of −3.5 °C. Due to the high applied voltages, burning phenomena could appear, and in order to avoid them, the samples were first pre-anodized under mild anodization electrochemical conditions at 80 V during 15 min to grow a protective alumina layer. The voltage was then swept at 0.05 V/s until reaching the HA regime at 110 V, while the temperature was reduced to −4.5 °C to avoid dielectric breakdown of the Al_2_O_3_ layer. After 60 s, the voltage was again swept at 0.05 V/s until reaching 140 V. The final HA step, at which the NAM effectively grows, lasted 90 min.

**Figure 8:**
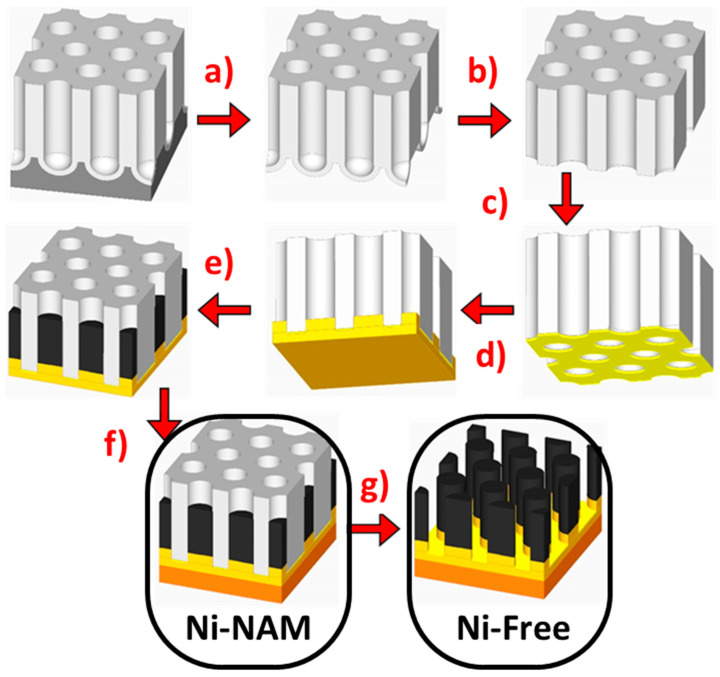
Schematic process flow for the fabrication of Ni nanowires embedded into the pre-patterned nanopores of the anodic aluminium oxide (AAO) template, starting from the hard anodization of the Al substrate. (**a**) Removal of Al substrate and alumina barrier layer. (**b**) Pore widening and barrier layer removal. (**c**) Deposition of Au electrode by sputtering and (**d**) subsequent electrodeposition of Au layer. (**e**) Electrochemical deposition of Ni nanowires inside the patterned pores of the alumina template. (**f**) Deposition of Cu base behind the Au layer (hereafter named as Ni-NAM sample). (**g**) Removal of AAO template (hereafter referred to as Ni-Free sample).

The workflow followed for the fabrication of Ni nanowires Is summarized in Figure 8. Starting from the HA-NAM template fabricated as explained above, the remaining Al at the bottom of the NAM is selectively etched away by means of a CuCl_2_ and HCl solution. To remove the barrier layer of Al_2_O_3_ at the bottom of the NAMs and the protective mild anodization layer at the top [58], the samples were submitted to a wet chemical etching step in phosphoric acid (5 wt.%, 30 °C) for 150 min. At this point, the pores of the NAM are opened at both sides, although a widening of the pore diameter is expected, as it is determined by scanning electron microscopy. Prior to the electrochemical deposition of Ni inside the pores of the NAM, a metallic seed layer at one side of such membrane must be placed. In this work, an Au seed layer has been sputtered and subsequently electrodeposited from a commercial electrolyte (Orosene), serving as a working electrode at one of the NAM surfaces.

To proceed with the electrochemical deposition of Ni, an electrolyte containing 30 g NiSO_4_·6H_2_O + 4.5 g NiCl_2_·6H_2_O + 4.5 g H_3_BO_3_ in 100 mL of aqueous solution was synthesized by adjusting the pH to 4.5. The potentiostatic electrodeposition of Ni nanowires consisted of a constant voltage of −1.2 V, during 2.5 min, measured versus an Ag/AgCl reference electrode.

### 4.2. Fabrication of Core/Shell Nanowires

Core/Shell Ni@(NiO,Ni(OH)_2_) nanowires were fabricated by a controlled electrochemical oxidation of Ni nanowires surface. In such a process, the nanowires surface must be exposed to an oxidative electrolyte. However, it is important to keep in mind that once the nanowires are electrodeposited inside the pores of the NAMs employed as patterned templates, their surface is chemically protected by the Al_2_O_3_ membrane, so it must be etched away. In order to provide enough mechanical stability to the sample (it would break otherwise once the Al_2_O_3_ template is removed), a thick Cu film is electrodeposited at the back side of the sample from a Cu-based electrolyte containing 4.5 g CuSO_4_ + 0.4 g Boric acid in 100 mL aqueous solution. Once the backside film is thick enough, the Al_2_O_3_ membrane can be selectively removed by wet chemical etching treatment in 1M NaOH for 90 min. This last process ends up with an array of nanowires only fixed at one side (Au/Cu film), which will be referred to as the “nanowire forest”.

The experimental device used to carry out both the cyclic voltammetry and the electrochemical oxidation of Ni nanowires consists of an electrochemical cell with three electrodes connected to an AMEL model 7050 potentiostat. These tests will serve to determine the values of potentials at which oxidation phase changes occur in nickel samples, thus being able to transform Ni^0^, to Ni^2+^, and to Ni^3+^. The electrolyte used in all cases is a 1M KOH solution [59].

### 4.3. Characterization Techniques

Morphological and compositional characterizations of NAMs templates and nanowire forests were performed using a Scanning Electron Microscopy device (SEM, JEOL 5600, Akishima, Tokyo, Japan) equipped with an energy dispersive X-ray (EDX) microanalysis system (INCA, Oxford Instruments, Abingdon, UK). Microstructural analysis of free-standing single nanowires was also studied using the Transmission Electron Microscope technique (TEM, JEOL-2100-EXII, Akishima, Tokyo, Japan). This system has been also employed to obtain Selected Area Electron Diffraction (SAED) patterns to evaluate the crystalline structure of NWs, suited with an EDX detector (X-MAX, Oxford Instruments, Abingdon, UK) and operating in scanning transmission mode for the EDX line-scan analysis of the layered core/shell heterostructure of Ni electrochemical oxidized nanowires.

X-ray photoelectron spectroscopy (XPS) surface analysis measurements were performed using a PHI Model 5701 MultiTechnique System with MultiChannel detector, with a nonmonochromatic X-ray source (Kα Mg = 1253.6 eV) at 15 kV and 300 W. The energy analyser worked on constant pass energy mode. Survey spectra were performed using 187.85 eV pass energy and 0.8 eV step energy and high-resolution spectra were taken with 29.35 eV pass energy and 0.125 eV step energy. Spatial resolution was achieved by using a small aperture size (720.0 µm). The reference energy value was C1s at 284.8 eV. Shirley type base lines and 70% Gaussian-30% Lorentizan curves were used for all mathematical fittings, carried out in Casa XPS software using Marquardt-Levenberg or Simplex methods [60]. Prior to the XPS characterization, the samples were cleaned with plenty of distilled water, to remove any surface contamination coming from organic sources.

Regarding the magnetic characterization of the samples, magnetic hysteresis loops measured along parallel and perpendicular directions of the applied magnetic field with respect to the nanowire axis, together with thermomagnetic curves characterization of Ni@(NiO,Ni(OH)_2_) nanowire arrays, were performed in a vibrating sample magnetometer (VSM, Versalab, Quantum Design Inc., San Diego, CA, USA), under applied magnetic field values up to ±3 T in the temperature range between 50 K and 400 K. First-order reversal curve (FORC) measurements and analysis, performed along the parallel direction, have also been employed in this study; basic details can be found elsewhere [48,61,62].

## 5. Conclusions

Ni nanowire arrays have been electrochemically deposited into the pores of hard anodic nanoporous alumina membranes, which were used as templates. As a result of that process, nanowires with a mean diameter of around 170 nm and a mean length of 4.2 µm have been obtained. After the selective wet chemical etching of Al_2_O_3_ membrane, an electrochemical treatment of Ni nanowires in 1M KOH electrolyte under an oxidative potential of −0.25 V has been carried out. As a result of the Ni nanowires surface oxidation to both NiO and Ni(OH)_2_ phases, two different magnetic behaviours have been encountered. On one side, a magnetic hardening of the nanowires (around 17% at room temperature) has been observed that might lead to an increase in the energy product of the FM/AFM system. This effect can be correlated to the formation of a low anisotropic NiO/Ni(OH)_2_ AFM shell. On the other hand, a strong EB effect has been also observed at relatively high temperatures (below 100 K) for nanoparticle systems, although a maximum magnetic field shift of −100 Oe has been obtained at the lowest measured temperature (50 K). The complex magnetic behaviour exhibited by these electrochemical oxidized Ni nanowires is also due to both the high structural irregularity and, as a consequence of that, the frustration of AFM coupling exhibited by the NiO and Ni(OH)_2_ crystalline phases that appear formed in the nanosized outer-shell layer of the nanowires, with a spin-glass-like magnetic behaviour. These results point out the potential of the purely electrochemical fabrication method of core (FM)/shell (AFM) Ni@(NiO,Ni(OH)_2_) nanowires, avoiding thermal oxidation steps completely. The completely avoidance of temperature treatments, often used to perform FM/AFM nanoparticles, is of great importance since it could be implemented in an integrative fabrication method of standard semiconductor technology.

## Data Availability

Data sharing is not applicable to this article.
